# Expression of Gag and Pol from reconstructed HERV-Fc1, associated with multiple sclerosis

**DOI:** 10.1186/1742-4690-8-S2-P56

**Published:** 2011-10-03

**Authors:** Kari K Nissen, Finn S Pedersen, Bjørn A Nexø

**Affiliations:** 1 Department of Biomedicine, Aarhus University, DK-8000 Aarhus C, Denmark; 2 Department of Molecular Biology and Genetics, Aarhus University, DK-8000 Aarhus C, Denmark

## Background

Though the etiology of Multiple Sclerosis (MS) is still obscure, Human Endogenous Retroviruses (HERVs) have long been suspected to be involved [1]. Functional studies have backed this theory, and recently our group provided more direct genetic evidence for association of MS with a provirus located on chromosome X, HERV-Fc1 [2]. An association with the retroviral restriction factor TRIM5 was also found.

The HERV-Fc1 sequence contains the general retrovirus structure of LTR-elements and the three genes *gag*, *pol* and *env.* The *env* gene seems intact with an open reading frame (ORF). The *gag* ORF is terminated by two stop codons, compared to the common single stop in exogenous retroviruses. The *pol* frame is interrupted by a frameshift mutation and a premature stop-codon; a polymorphic C-repeat in the *pol* gene represents a second frameshift in some persons.

HERV-Fc1 is only sparsely characterized, and we therefore aimed to investigate this provirus, especially in relation to its potential involvement in autoimmunity.

## Materials and methods

The HERV-Fc1 *gag* and *gag-pol* genes were cloned into expression vectors in a CMV-promoter context. Due to lack of commercial antibodies, the genes were fused to C-terminal 6xHis-tags. For one set of vectors, the *pol* reading frame was restored by point mutations of the nucleotides disrupting ORF. All clones were fully sequenced to ensure correct sequence, before cellular expression. Protein expression was determined by Western blotting and immunohistochemistry.

## Results

Expression of both Gag and GagPol polyproteins was detected. Both high molecular weight (>140kDa) GagPol and lower (~40kDa) presumed íntegrase protein was found. A longer stretch of the 5'UTR region preceding *gag* was necessary for expression; no protein expression was obtained when only including a few nucleotides before the ORF. Inclusion of the 5'LTR region inhibited the expression.

Upon expression, pelletable Fc1 Gag could be detected in the culturing media. This cellular exclusion seem specific, since GFP-6xHis fusion-protein expressed from a similar vector and endogenous Beta-tubulin could not be detected. Reverse Transcriptase activity has so far not been detected from the *gag-pol* constructs.

## Conclusion

The HERV-Fc1 *gag* gene has potential for immediate expression; this expression is dependent on the 5'UTR region of *gag.* Expression of Fc1 GagPol polyprotein could be achieved upon only three point mutations, with read-through of one stop-codon between *gag* and *pol.* Cellular exclusion of Gag suggests particle formation and export.

These vector constructs can be used in future characterization of HERV-Fc1, e.g. tropism determined by host restriction factors, drug sensitivity etc.
